# Report of the Medical Officer of the Education Committee of the London County Council

**Published:** 1908-01-18

**Authors:** 


### Public Health and Hygiene.

### REPORT OF THE MEDICAL OFFICER OF THE EDUCATION COMMITTEE OF THE LONDON COUNTY COUNCIL.

We have read with considerable interest the report recently published of the Medical Officer (Education) to the London County Council. The document is especially valuable at the present time, when education authorities throughout the country are under statutory obligation to make provision for medical inspection, and for attending to the health and physical condition of the children educated in public elementary schools. The report embraces a wide variety of conditions which have been investigated by the medical officers employed by the London County Council, and its perusal cannot fail to impress the reader with the vast amount of illness and physical shortcoming affecting the children attending the public elementary schools in the county of London. We could wish that the report had been presented in more readable form, for the importance of the matter dealt with would have justified more care in setting down in clear English the meaning which the writer wished to convey. Some sections abound in grammatical errors, and in large part the report is written in a loose, confused style, characterised by a lack of precise statement and a profusion of ambiguous, structureless sentences, which convey nothing, owing to an ambitious attempt to say too much. Several of the tables, for lack of adequate descriptive context or headings, are barely intelligible, and the effect of these blemishes is an impression that the value of the experience gained in the schools has suffered from a hasty presentment of the results. Dr. Kerr has brought to light, however, some valuable data based on his experience of the medical inspection of school children in London. It is possible to construct from his report evidence of an overwhelming character as to the need for serious investigation into the health and physical condition of children attending public elementary schools. Of the supreme importance of organising measures of a remedial nature on lines similar to those suggested in the report, there can be no question, and what is most clearly conveyed is that a very large proportion of the children are in need of medical care. Different schools yield different proportions of scholars suffering from physical defects. In infants' schools these proportions were found to vary between 9 per cent. and 26 per cent., while the proportion of particular defects to defects of all kinds may be gathered from the following table taken from his report:—

| | Percentage of all the children with defects having particular defect. |
|---|---|
| Vision ... ... ... ... ... | 19.8 |
| Hearing ... ... ... ... ... | 7.7 |
| Physique ... ... ... ... ... | 27.5 |
| Speech defects ... ... ... ... | 5.25 |
| Mental defects ... ... ... ... | 9.75 |
| Tonsils and adenoids ... ... ... | 31.5 |
| Enlarged cervical glands ... ... ... | 14.5 |
| Discharge from ears ... ... ... | 3.5 |

Dental defects appear to be of a most widespread and serious character, and the magnitude of the problem of dealing with them may be gathered from the following statement of Dr. Kerr: "At present there is not hospital accommodation in London for more than about 200 cases weekly—say, 10,000 annually—but at least ten times this accommodation would be required to relieve even a part of the children whom school dentists would select as requiring it."

Deformities due to lack of, or to improper form of, exercise, to defective nutrition, or to habitual vicious posture, appear to be exceedingly common, especially among girls attending elementary schools, and investigation in respect of these defects has shown abnormalities of spinal curvature to be present in as high a proportion as 64 per cent. of the scholars attending school. Pulmonary tuberculosis, as might be expected, does not rank as a disease seriously affecting school children, and only about 0.5 per cent. of the scholars examined exhibited physical signs such as would warrant the diagnosis of this condition. Adenoids and morbid conditions of the tonsils, on the other hand appear to be extremely prevalent, and when one remembers the serious sequelæ of these affections the importance of curative measures as a branch of preventive medicine becomes strikingly apparent. A careful reading of the report, however, does not lead one to endorse the view of the writer that school hygiene is "a highly specialised branch of public health, which the ordinary sanitarian cannot be expected to follow in its fulness, and which requires officers experienced in the technique and specially trained to do justice to the children and to the public interests" (*sic*).

Dr. Kerr appears to have a poorer opinion of the "ordinary sanitarian," by whom, we presume, he means the medical officer of health, than he has of the school nurse or school teacher. For he says, "It is quite absurd to say that a nurse cannot recognise ringworm. This is a matter of experience, and we find the nurse's judgment quite reliable." Again he says, under the heading of "Tonsils and Adenoids": "No teacher is fit to have charge of a class of young children *who cannot give a pretty fair opinion* as to whether a child is suffering seriously in this respect or not."

And again he says: "Our experience shows the usual official sanitary idea of results in measles prevention by school or class closure is mere imagination, and that closure, applied in the only successful manner, at the occurrence of a first case, postpones measles for such a short time that the interference with school work, except under special conditions, is hardly justified, and that for any effective results *trained teachers capable of detecting the first case of measles are an absolute necessity*" (the italics are ours).

"The ordinary sanitarian," who stands for "the usual official sanitary idea," in this country is a medical man who has not hitherto detached himself from the profession with which he is proud to be identified. And we think it will be a misfortune to medicine, and to the people of this country, should the further differentiation of practice—specialisation, as it is called—lead to such utter professional disintegration as is foreshadowed in the sentences we have just quoted. Nor, as we have said, do we find from the report that the nature of the medical work done in the public elementary schools in London is of that ultra-specialised type that it has passed beyond the competence of the ordinary practitioner of medicine. The conditions from which the children suffer are just those unfortunately common diseases which every general practitioner is dealing with every day of his life. The diagnosis of the nature of these conditions is what medical men happen specifically to have been trained to make, and for the most part prevention or treatment has failed, mainly because it has never been undertaken at all, and partly because of the impossible conditions of practice where the poorer class of children are concerned.

We quite agree with Dr. Kerr that treatment will have to be considered as a public concern, and that school clinics will require to be established to deal with those cases which at present are not seen at all by medical men. And we say, further, that provision must be made to remove the hideous anomaly of thousands of children suffering from preventable and curable diseases at present utterly neglected, while millions of pounds are being spent in an attempt to educate the victims of these disabling conditions.

It is the great merit of the Education (Administrative Provisions) Act of 1907 that local authorities are now empowered to make such arrangements as may be sanctioned for the removal of this anomaly. We think, however, that in the organisation of this work, while it will certainly be desirable to enlist the co-operation of the teacher and the nurse, it will be a profound mistake to confuse the proper functions of either.

A teacher should be as capable as an ordinary mother of judging when a child is ill and requires the care and skill of the medical practitioner, and a nurse is properly exercising her training in seeing that the treatment prescribed by the medical man is carried out as he instructs. But it is an inexcusable blunder to confound their duties with those of the doctor, and to imagine such a vain thing as teachers and nurses diagnosing diseases which the trained practitioner, if he knows his business, will approach with becoming modesty in the full consciousness of his liability to error.

The *medical* inspection of school children, organised in accordance with the requirements of the Board of Education, as set out in the recently published Memorandum, we may confidently assume will not be associated with this deplorable mistake. Medical officers of health have had a long experience of the employment of lay assistance in carrying out their duties, and this experience has taught the few who have needed the lesson that diagnosis is the function of the medical practitioner alone.

Notwithstanding the many defects in the form of the report, there is in the material, proof of most creditable work. It is not too much to say that were there no other evidence than is presented in its pages it would be sufficient warrant for the steps taken by the Board of Education to ensure that the blot on the system of national education, of which these results are in some degree a measure, shall in the future be removed.

